# The Effect of Polymeric Nanofibers Used for 3D-Printed Scaffolds on Cellular Activity in Tissue Engineering: A Review

**DOI:** 10.3390/ijms24119464

**Published:** 2023-05-30

**Authors:** Davood Kharaghani, Elmira Kaffashsaei, Md. Kaiser Haider, Ick Soo Kim

**Affiliations:** 1 Department of Calcified Tissue Biology, Graduate School of Biomedical and Health Sciences, Hiroshima University, 1-2-3 Kasumi, Minami-Ku, Hiroshima 734-8553, Japan; 2 Department of Bioproducts and Biosystems Engineering, University of Minnesota, 2004 Folwell Avenue, St. Paul, MN 55108, USA; 3 Nano Fusion Technology Research Group, Institute for Fiber Engineering (IFES), Interdisciplinary Cluster for Cutting Edge Research (ICCER), Shinshu University, Tokida 3-15-1, Ueda 386-8567, Japan; 22hs108f@shinshu-u.ac.jp (M.K.H.); kim@shinshu-u.ac.jp (I.S.K.)

**Keywords:** 3D bioprinting, nanofibers, tissue engineering, reinforce hydrogels

## Abstract

Promising scaffolds for developing advanced tissue engineering architectures have emerged in recent years through the use of nanofibers and 3D printing technologies. Despite this, structural integrity and cell proliferation are highlighted as fundamental challenges for design scaffolds and future prospects. As a biomimetic scaffold, the nanofiber-reinforced hydrogels demonstrated a better compressive modulus and cell growth. Our review focuses on recent promising advances in the development of 3D-printed hydrogels containing polymeric nanofibers that can improve cell-material interaction in biomedical applications. Moreover, an effort has been made to induce studies with diverse types of scaffolds for various cells. Additionally, we discuss the challenges and future prospects of 3D-bioprinted reinforced hydrogels with nanofibers in the medical field, as well as high-performance bioinks.

## 1. Introduction

In the area of prosthetics, transplantation, and stem cell therapy, tissue engineering is a promising technique for the recovery of damaged tissues and organs [1]. Tissue engineering is generally a multidisciplinary field involving cells, bioactive compounds, and scaffolds [2]. As scaffolds have been developed over the past few years, three-dimensional (3D) porous scaffolds, have been highlighted for their ability to maintain nutrients while transporting metabolic wastes and mimicking the architecture of tissues and organs [3]. The 3D bioprinting technology has emerged as a promising method for engineering complex 3D structures through the layer-by-layer deposition of cell-free or cell-laden bioinks [4]. Among the trials, the dilemma is that too soft bioinks cannot be stacked in a 3D structure with the desired shape [5]. To solve this problem, several studies have been conducted using nanofibers, which will be reviewed in this paper. Since natural tissues are composed of fibrous extracellular matrix (ECM) networks to maintain their toughness properties [6], recent studies have focused on 3D-printed hydrogels reinforced with nanofibers. One of the major challenges in the field of tissue engineering is to develop scaffolds that mimic the architecture of tissues at the nanoscale [7]. Nanofibers have greatly enhanced the scope for fabricating scaffolds that are able to address these challenges. The availability of a wide variety of natural and synthetic nanofibers has increased the potential for developing scaffolds containing fibers for tissue engineering. Nanofibrous scaffolds serve as an excellent framework for cell adhesion [8], proliferation [9], and differentiation [10].

Despite the numerous methods that have emerged for the fabrication of nanofibers, electrospinning has been highlighted as a simple, inexpensive, versatile, and evolving process for generating nanofibers from various polymers. Electrospinning involves an electrodynamic process where a liquid droplet is elongated and stretched into a continuous fiber through an electric field. These fibers typically range in diameter from tens of nanometers to a few micrometers [11]. In addition to being involved in drug delivery systems [12], nanofibers have been employed for the regeneration of various tissues, including wound dressing [13], bone [14], cardiovascular [15], and blood vessels [16]. For 3D scaffold design, both the material and fabrication method should be carefully considered, as the composition and structure of the ECM regulate the scaffold network’s structure and biomechanical properties.

In recent years, several tissue-specific bioinks reinforced with nanofibers have been introduced commercially. As discussed in the associated literature, there is a wide range of possible uses of nanofibers in the design of tissue-specific bioinks. This review focuses on the interaction between cells and materials by using readily available 3D-printed hydrogels in composition with nanofibers for various types of cells. As well as reviewing and comparing their individual effects on biological systems, the advantages, limitations, and future prospects of these technologies are discussed.

## 2. Tissue Engineering

**Figure 1:**
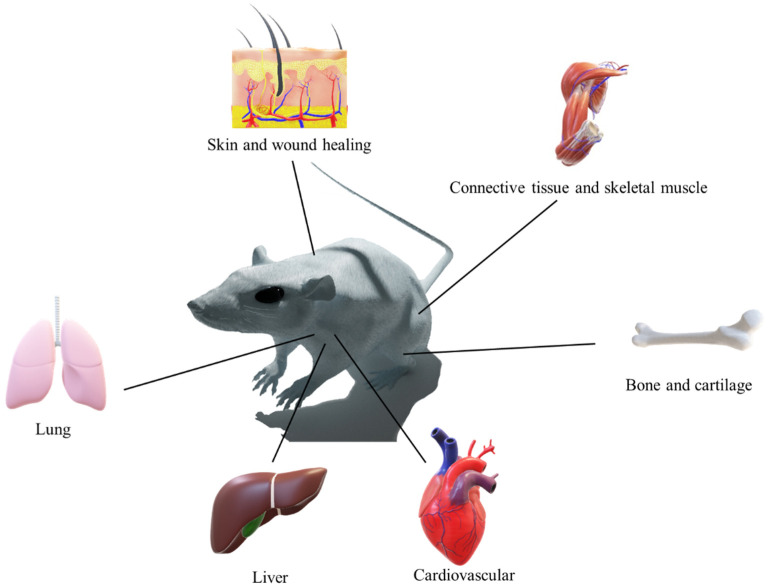
Biomedical application of the combination of 3D printing and polymeric nanofibers.

Tissue engineering is an approach to regenerating damaged or dead tissue using scaffolds. These scaffolds are highly porous biomaterials that act as a tissue regeneration template. The major focus of tissue engineering is to replicate or restore the lost function of failed organ/damaged tissue by integrating cells and biomaterials, reviewed perfectly elsewhere [17]. As illustrated in Figure 1, polymeric nanofibers are used in 3D-printed scaffolds for regeneration of various organs and tissues.

### 2.1. Bioprinting: Drawbacks and Advantages

The bioprinting technique involves the robotic deposition of cells and/or biomaterials into custom-shaped and patterned 3D scaffolds to mimic complex tissue architectures. As part of the trials, bioinks should meet the following requirements: be highly printable while providing a robust and biocompatible microenvironment. Sadly, most hydrogel bioinks have not been able to meet these requirements and are mechanically weak due to their heterogeneously crosslinked network and lack of energy dissipation mechanisms [18]. Even though advanced bioinks must exhibit high print fidelity, be biocompatible, as well as possess superior mechanical properties, there are some limitations to their application performance, including low resolution with 3D printing and uncontrollable shape with products. Therefore, new ideas that combine 3D printing and nanofibers have been explored in order to maximize their strengths and mitigate their weaknesses, thus providing an efficient and effective way to fabricate novel scaffolds [19].

### 2.2. Cardiovascular

**Figure 2:**
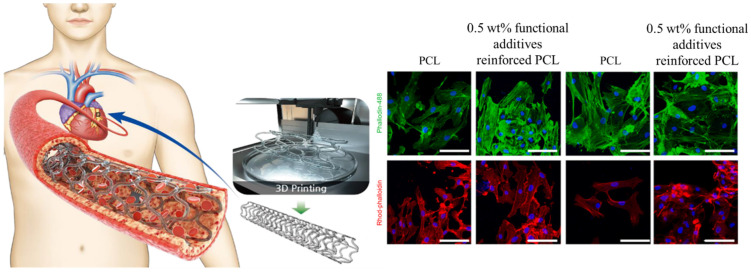
Cellular activity of 3D printed cardiovascular PCL scaffold reinforced by gold particles incorporated carbon nanofibers. Reproduced with permission [21]. Copyright Elsevier 2023.

Globally, cardiovascular diseases (CVDs) account for the majority of deaths [20]. A number of studies have been conducted to investigate the surface morphology and the effects of geometric and fabrication factors on the surface of 3D-printed cardiovascular scaffolds. Sulfur-doped carbon nanofibers in composition with gold nanoparticles have been used [21] as an additive for polycaprolactone bioink and 3D printing techniques employed to fabricate the scaffold as shown in Figure 2. In vitro, studies were conducted using human umbilical vein endothelial cells (HUVECs) and vascular smooth muscle cells (VSMCs). A cytocompatibility study revealed that the viability of HUVEC and VSMC on the scaffold with or without NIR irradiation ranged between 70% and 90%, compared with control cells. Moreover, a slightly higher proliferation rate was observed in functional additives incorporating bioink, which has been linked to a reduction in inherent hydrophobicity and an improvement in conductivity. Moreover, both HUVECs and VSMCs adhered to and proliferated on the surface of prepared scaffolds.

In independent research [22], a hierarchically organized, anisotropic and conductive scaffold has been fabricated using electrospinning and melt-spinning techniques. Initially, aligned PCL nanofibers were fabricated using electrospinning, and then patterned microgrooves were fabricated with melt electrospinning on top of aligned PCL mesh coated with Au. To study the effect of scaffolds’ topographic cues on cell behaviors, H9c2 cells were seeded on scaffolds with different mesh. Upon culture of H9c2 cells in differentiation medium for 5 days, the staining of myosin heavy chain, a protein necessary for the formation of myotubes, is observed. A quantitative analysis of cell orientation revealed that 74.6% of myoblasts were aligned with nanofibers on parallel aligned scaffolds, slightly higher than the pure aligned PCL mesh (71.4%). A further study indicated that scaffolds coated with Au nanoparticles significantly promote the elongation of myotubes due to the enhancement of scaffold conductivity.

Moreover, PCL nanofiber combined with rotary bioprinting [23] for the fabrication of small-diameter vessels with endothelium and smooth muscle cells. In order to achieve this, electrospun PCL tubes were coated with different concentrations of methacrylate gelatin containing smooth muscle cells using bioprinting techniques. In vitro, results indicated that HUVECs and SMCs were successfully stratified in the printing process with a good survival rate and over 90% cell viability. CD31 and α-SMA immunofluorescence staining indicated that HUVECs trimly covered the interior surface of artificial blood vessels and exterior 3D printed scaffold blended with SMCs.

Intimal hyperplasia and restenosis caused by excessive SMC proliferation are the main factors for the failure of stent implantation. Chengjin Wang and colleagues [24] proposed to development of bioresorbable stents made from PCL using 3D printing that coated with dipyridamole (DP) loaded poly(D, L-lactide) (PDLLA) nanofibers. Their research compared three different scaffolds, including Stent/PDLLA nanofibers/DP, Stent/PDLLA nanofibers, and stent using rat SMCs and HUVECs. SMCs and HUVECs seeded onto PDLLA nanofibers showed significantly higher cell proliferation rates than those seeded on stents. Although the purpose of this research was to inhibit the proliferation of rat SMCs with DP, the SMCs proliferated over the stent/PDLLA nanofibers when compared with the stent without nanofibers, indicating that PDLLA nanofibers enhanced cell proliferation.

### 2.3. Cartilage

A meniscal injury to the knee joint is more likely to result in a permanent alteration of the knee joint as a result of the morphological mismatch and substantial loss of meniscal tissue. A number of additives have been used in the development of meniscus, including cellulose nanofibers (CNFs). To improve printability, CNF was mixed with gelatin-alginate, and the viability and metabolic activity of the fibrochondrocyte cells were evaluated. At day 14, there was a significant decrease in the metabolic activity of encapsulated cells [25]. Unfortunately, the mentioned research did not examine the effect of CNF nanofibers on cellular functions.

Towards bioinspired meniscus regeneration [26], a scaffold composed of bioprinted cells infused with type I collagen reinforced by multilayers of biomimetic PCL/carbon nanotubes (CNT) was developed by Thaiago and colleagues. It is the only test available for this research that uses isolated rat MSCs to determine the viability of the cells. Bioinks containing type I collagen and isolated rat MSCs were used (Lifeink 200, Advanced BioMatrix, CA, USA) for this application. A quantitative analysis of cell viability demonstrated that rat MSCs within scaffolds, whether with or without nanofiber membranes, retained greater than 80% viability after 7 days.

M.Rampichova and colleagues [27] developed a two-step method for preparing a composite scaffold of 3D printed grids and electrospun nanofibers. The first step involved producing the non-patterned PCL electrospun nanofibers, patterned PCL nanofibers, and 3D printing of microfibrous grid using PCL. The second step involved assembling the products from the first stem using glue. In order to assess cell-composite interaction, isolated chondrocytes from the condyle of a pig’s femur were used. The in vitro evaluations have demonstrated that chondrocyte cells proliferate better on patterned electrospun PCL nanofibers than on composite 3D printed scaffolds with patterned or non-patterned scaffolds. According to the authors, the higher energy consumption for cell spreading and migration on the 3D scaffolds caused a decrease in proliferation in comparison with neat PCL nanofibers. Although the number of cells on neat nanofibers continued to increase, most metabolic activity appeared to be associated with cell proliferation. Moreover, the expression of the Type II collagen gene was delayed for 3D printed scaffolds containing patterned and non-patterned nanofibers, while the highest expression was associated with non-patterned neat PCL nanofibers on day 14.

In independent research [28], human mesenchymal stem cells (hMSCs) were used to prepare a cell-laden bioink for cartilage regeneration. In this study, hMSCs were blended with hydroxy butyl chitosan (HBC) hydrogel and PCL short nanofibers. In comparison to the HBS hydrogel, the hMSCs exhibited a higher growth rate in the HBC-PCL short nanofiber. Additionally, immunochemical staining of collagen II in HBS-short PCL nanofibers containing hMSCs showed superior expression of Type II collagen.

### 2.4. Liver

**Figure 3:**
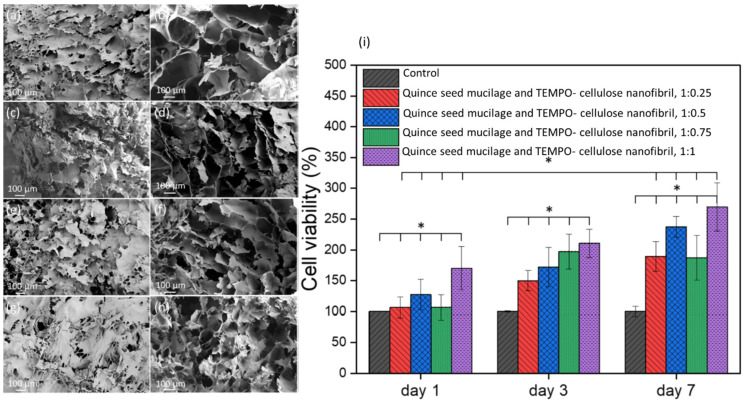
SEM images from the surface and cross-section area of the lyophilized samples containing TEMPO- cellulose nanofibril and Quince seed mucilage with the ratio of 1:0.25 (**a**,**b**), 1:0.5 (**c**,**d**), 1:0.75 (**e**,**f**), and 1:1 (**g**,**h**). Cell viability of HepG2 cells in the presence of hybrid hydrogels after 1, 3, and 7 days of incubation (**i**). * *p* < 0.05. Reproduced from open access reference [29].

Planet-based hydrogels have attracted great attention in biomedical sciences. A new viscoelastic ink [29] based on quince seed mucilage and cellulose nanofibrils and extruded into 3D lattices was developed. To assess the cytocompatibility of the samples with different ratios of cellulose nanofibrils, HepG2 cells were cultured, and the cell proliferation assay was the only biological test used in this study. It was revealed in the results of the study that increasing the concentration of cellulose nanofibrils enhanced the proliferation of HepG2 cells (Figure 3).

### 2.5. Bone

Bioabsorbable polymers, such as polylactic acid (PLA), are widely used for implants, such as pins, plates, and screws, which degrade in the body primarily by hydrolysis as the bone union process progresses [30]. A 3D-printed screw made from PLA was coated with polyvinyl alcohol (PVA), which contains varying concentrations of nanohydroxyapatite (nHA). The cytotoxicity and adhesion behaviors of samples were tested in vitro using MC3T3-E1 cells. The proliferation and adhesion assays indicated that with increasing nHA concentration, cell proliferation increased significantly. In the reviewed research, differentiation and mineralization of MC3T3-E1 cells were not examined [31].

Nanocellulose pellicle was produced as a byproduct during the symbiotic culture of bacteria and yeast in kombucha. Mamatha and colleagues used fibrillated nanocellulose to prepare the scaffold in a 3D bioprinting process. The prepared samples were tested for cytocompatibility using human adult dermal fibroblasts (HADF) and MC3T3-E1 cells. In comparison with tissue culture palate as a control, the viability of MC3T3-E1 cells did not demonstrate any cytotoxicity against the HADF, and results indicated that the cells proliferated with a ratio of 80% [32].

Regenerative medicine requires the development of a biomimetic cell microenvironment closely resembling native tissue. Dilshan and colleagues [33] prepared a novel meniscus tissue scaffold using a 3D-printed frame made of poly (lactic acid) (PLA) and random or aligned PCL and collagen nanofibers embedded between two frames. MG-63 cells were used to characterize scaffolds in vitro. According to the results, MG-63 cells adhered to and proliferated on the hybrid scaffold, and topological/biophysical cues can be incorporated into a nanofiber matrix to influence cell growth.

### 2.6. Lung

Researchers have investigated the relationship between airborne transmission, mold exposure, and inflammatory airway diseases. Reports indicate that deoxynivalenol (DON), a secondary metabolite primarily produced by Fusarium fungi, is the principal risk factor for farm workers [34,35]. A cell electrochemical sensor based on 3D bioprinting was designed [36] to evaluate the individual toxicity of DON and its derivatives. Bioink was prepared by mixing culture medium, carbon nanofibers, gold nanoparticles, GelMA at different concentrations ranging from 5% to 15%, and A549 cells. As shown by the live/dead staining method, the survival rate of cells was significantly reduced by increasing the concentration of GelMA hydrogel on scaffolds with 5–7.5% of GelMA hydrogel.

A bioink containing regenerated silk fibroin and 2,26,6-tetramethylpiperidine-1-oxyl (TEMPO) oxidized bacterial cellulose (OBC) nanofibrils was developed for 3D printing lung tissue scaffolds [37]. In vitro, evaluation of the viability and morphology of the prepared scaffold was conducted using lung epithelial stem cells isolated from human lungs. Due to the presence of oxidized bacterial cellulose nanofibrils on the surface of the printed scaffolds for three days, the MTT results indicate that LESCs grew faster on the scaffolds than on the tissue culture plates. As a result, the proliferation rate decreased after day 3 compared to tissue culture plates for an unknown reason.

### 2.7. Connective Tissue

Due to the fact that bacterial cellulose fibers are in the range of collagen fibril diameters in most tissues, bacterial cellulose has attracted the greatest amount of attention in tissue engineering applications. The bioink was developed using bacterial cellulose nanofibers mixed with silk fibroin and gelatin at different concentrations [38]. In vitro biocompatibility and cell infiltration of printed scaffolds were assessed using L929 cells. On the composite scaffolds containing the highest concentration of bacterial cellulose (1.4 wt.%), more live cells (viability greater than 80%) were observed, and cells proliferated only on those scaffolds containing the highest concentration of bacterial cellulose nanofibers. As suggested by the author, this could be due to the obvious interconnected porous and fibrous structure of scaffolds and the hierarchical pores ranging from nanoscale to macroscale.

**Figure 4:**
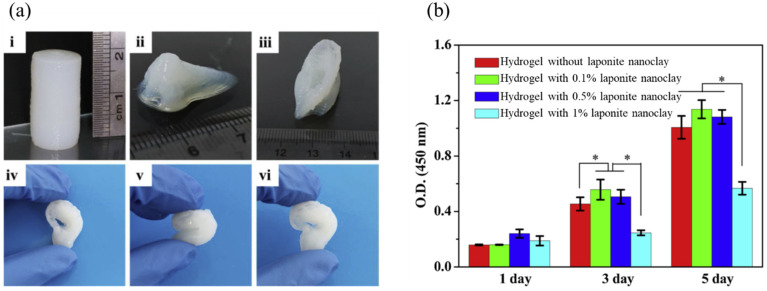
The photo of printed complex structures from TEMPO-oxidized bacterial cellulose/alginate hydrogel containing 0.5% laponite nanoclay (**a**) cylinder (**i**), nose (**ii**), ear (**iii**), and the photo of squeezing and recovery of the printed ear (**iv**–**vi**). The cell proliferation of L929 on the surface of hydrogels on days 1, 3, and 5 (**b**) * *p* < 0.05. Reproduced with permission [39]. Copyright Elsevier 2023.

L929 cells were also used for the evaluation of a 3D printed scaffold [39] made by TEMPO-oxidized bacterial cellulose and sodium alginate mixed with different concentrations of laponite nanoclay (0–1 wt.%) as shown in Figure 4. A cellular evaluation of the scaffolds revealed that on the third day of culturing, cell numbers decreased by increasing the laponite nanoclay concentration to 1 wt.% nanoclay adhesion to the cell surface and restriction of cell function.

An ink with a dual crosslinking capability composed of poly(ethylene glycol) and different concentrations of (TEMPO)-oxidized nanocellulose fibers were developed [40]. The viability of the cells was evaluated following extraction of the prepared hydrogel (containing 3 wt.% nanocellulose fibers) in DMEM for 24 h. The result indicates that the cell viability percentage of the ink hydrogel was 90.3 ± 9.5%. On the scaffold surface, the L929 cells exhibited elongated cell structures and cell adhesions.

Matteo and colleagues prepared ink using pectin and (TEMPO)-oxidized nanocellulose fibers [41]. Inks containing 2.5% pectin concentration demonstrated the greatest printing accuracy, and the effects of (TEMPO)-oxidized nanocellulose fibers concentration on the viability of L929 cells encapsulated in the hydrogel were investigated. Despite the decrease in cell viability for 1.5% *w*/*v* (TEMPO)-oxidized nanocellulose fibers, both bulk and 3D printed cell-laden scaffolds displayed greater than 80% viability, indicating that composition affects cell viability but not 3D structure.

The development of a hybrid 3D-printed scaffold that contains PLA frames and a layer of electrospun PCL nanofibers was achieved [42]. Cells from human fibroblasts were used for the in vitro cell culture study to compare the hybrid scaffold containing random and aligned fibers. The study results indicate that cells proliferate well on scaffolds containing nanofibers with random morphologies. The effect of fiber diameter on cell adhesion and proliferation was also determined by 20% PCL, which caused the fiber diameter to increase to over one micron, reducing the surface area available for cellular attachment, and 10% PCL, which increased the fiber diameter, decreasing the directional growth of cells, thus increasing porosity. Based on these results, there is a nonlinear relationship between fiber diameter, density, and cell growth.

### 2.8. Wound Healing and Skin Regeneration

Besides mimicking the structure of the extracellular matrix, nanofibers improve the printability of bioinks. An evaluation of the gelMA/PLGA-nanofiber hydrogel using NIH3T3 fibroblasts was conducted. The reported results indicated that the effect of PLGA-nanofibers on fibroblast viability in bioprinted gelMA was not significant. However, the hydrophilicity of PLGA fragments facilitates the spreading of cells in the gelMa hydrogel. Although F-actin fluorescent staining showed that cells in gelMa hydrogel remained in a spherical cluster after 3 days, the cells in the gelMA/PLGA-nanofibers hydrogel showed a partial stretch [43]. 

Moreover, NIH3T3 fibroblast cells were used to evaluate bioink composed of cellulose nanofibrils (prepared using the TEMPO oxidation method) and waterborne polyurethane. Cell viability (over 14 days) in the scaffold showed a proliferation rate of more than five times that of the initial number (537%), with a large aggregation of cells among the fibers and micropores [44]. The highly crystalline structure of silk, as well as its well-documented biocompatibility, makes it a promising material for use as reinforcement material and in the construction of functionalized composite scaffolds. The 3D printed scaffold with silk fibroin fiber and chitosan bioink was evaluated using human skin fibroblasts, and the results were compared with composites containing silk powder and silk microfibers. In an unexpected finding, on day 7 of cell proliferation, the composite containing chitosan silk fiber demonstrated a significantly better cell response than the composition containing chitosan powder or silk microfibers. The results indicated that the fibroblast cells on nanofiber-containing scaffolds were much more elongated, and cytoskeletal stress fibers could be seen, indicating a distinct cell response [45].

Bacterial cellulose nanofibers are an attractive biopolymer that has been used vastly for wound healing and skin regeneration. Acid treatment to hydrolase fibers was used to control the process for extrusion and shape formation of bacterial cellulose for 3D printing. The analysis of pure and partially hydrolyzed bacterial cellulose was conducted using adult human dermal fibroblast cells. In both samples, viability was approximately 80%, and cell adhesion was acceptable [32].

There are a number of challenging biomedical applications that can be addressed using electroconductive biomaterials. A hybrid printable composite containing alginate and gelatin hydrogels filled with different concentrations of carbon nanofibers was developed by Serafin and colleagues. The biocompatibility of prepared 3D-printed composites was evaluated using NIH3T3 cells. Although the cell viability for all groups ranged above 88%, the proliferation of cells was highest for the alginate-gelatin without carbon nanofibers [46].

## 3. Principal Challenges and Future Perspectives

In several research studies, it has been demonstrated that nanofibrous scaffolds are useful in tissue engineering, particularly when combined with hydrogels as bioinks for bio 3D printing. The development of 3D printed platforms for tissue engineering using nanofibers and hydrogels in mimicry of the ECM may benefit from the ability to reinforce and promote the biomimetic properties of the scaffold. In order to address fundamental issues, such as the selection of the most advantageous nanofibers to be used in tissue-specific platforms, further research will be required. A significant early step in this process will be the development of tissue-specific nanofibers compatible with the microenvironment during the regeneration process. Furthermore, we believe that future approaches that incorporate nanofibers may employ multifunctional nano scaffolds incorporating nanomaterials. Optimizing the composition ratio, morphology, and appropriate polymers based on the instability of tissue-specific scaffolds, improving cellular activity, and preventing systemic rejection, are all issues that need to be considered.

## Data Availability

Not applicable.
